# Predictive Maintenance with Sensor Data Analytics on a Raspberry Pi-Based Experimental Platform

**DOI:** 10.3390/s19183884

**Published:** 2019-09-09

**Authors:** Shang-Yi Chuang, Nilima Sahoo, Hung-Wei Lin, Yeong-Hwa Chang

**Affiliations:** 1 Department of Electrical Engineering, Chang Gung University, Taoyuan City 333, Taiwan; m0421011@cgu.edu.tw (S.-Y.C.); nilimasahoo263@gmail.com (N.S.); 2 Department of Electrical Engineering, Lee-Ming Institute of Technology, New Taipei City 243, Taiwan; hwlin@mail.lit.edu.tw; 3 Department of Electrical Engineering, Ming Chi University of Technology, New Taipei City 243, Taiwan

**Keywords:** predictive maintenance, data analysis, environment sensing, Raspberry Pi, PIC

## Abstract

Predictive maintenance techniques can determine the conditions of equipment in order to evaluate when maintenance should be performed. Thus, it minimizes the unexpected device downtime, lowers the maintenance costs, extends equipment lifecycle, etc. Therefore, this article developed a predictive maintenance mechanism with the construction of a test platform and data analysis along with machine learning. The information transmission of sensors was based on Raspberry Pi via the TCP/IP (Transmission Control Protocol/Internet Protocol) communication protocol. The sensors used for environmental sensing were implemented on the programmable interface controller and the data were stored in time sequence. A statistical analysis software platform was adopted for data preprocessing, modelling, and prediction to provide necessary maintenance decision. Using multivariate analysis users can obtain more information about the equipment’s status, and the administrator can also determine the operational situation before unexpected device anomalies. The developed modules are decisively helpful in preventing unpredictable losses, thus improving the quality of services.

## 1. Introduction

Interest in innovative processes such as predictive maintenance is growing rapidly. In recent years, due to the fierce competition among manufacturing industries, an increasing number of manufacturing companies are gradually expanding their business by selling products and providing good service to maintain a leading position. The role of maintenance plays a vital role in the path towards sustainable manufacturing. Maintaining the scheduling of service resources is a key step in the provision of product services. Reducing maintenance costs and improving service quality has become an important problem for industries. For example, agricultural equipment is on a tight schedule during the harvesting season; in this case, failure may cause a large loss in capacity. If the failure of the machine can be predicted in time, as per the failure prediction information, the company can provide requisite service to solve the problem reasonably, thus the possibility of failure can be eliminated which ensures the guarantee of a working machine. Therefore, predictive maintenance is considered an appropriate approach to ensure the healthy functioning of a device as it allows preventative detection of failure and avoidance of the breakdown of a device. 

Most maintenance methods use regular maintenance to maintain the normal operation of a device, but the cost of the parts and oils that are replaced during maintenance cannot be underestimated. According to the International Society of Automation, the cost of machine downtime is very high and approximately $647 billion is lost globally each year [1]. Regarding the importance of the impact of maintenance on equipment [2,3,4,5], many people think that regular maintenance is a profit-making strategy for manufacturers and a waste of time for users as long as the machine is in good condition; thus, it is considered non-essential. However, it may affect the integrity and durability of the device. If the device suddenly fails or stops working, it will cost more to fix. For example, in the case of locomotives, if the oil is not changed regularly, then in future, it may cause permanent damage to the engine which will cost more. Even if the user has to maintain the equipment according to the rules set by the manufacturer, sometimes there are still malfunctions which occur unpredictably and which cannot be prevented. Therefore, if a predictive maintenance technique is used, then permanent damage of the device may be prevented. The advantage of predictive maintenance is that it does not require manual intervention to determine when the equipment should be maintained [6,7,8]. Although experience is also an important factor, it cannot be considered as completely accurate. However, in predictive maintenance, the system will make decisions and schedule the best time for maintenance on its own according to the requirements. Big data analysis and machine learning can aid in predicting the period when devices are likely to become damaged and also the best time to replace parts before failures occur [9,10]. 

Statistical analysis software converts and analyzes a large amount of data accumulated by the long-term sensing module. It determines a correlation between the data and then sets the prediction. It is also possible to find out the settings that affect the environment and schedule a best maintenance time to reduce the number of maintenance sessions which will ultimately save time and unnecessary expense without affecting the normal operation of the device. Then, through the prediction software, it can predict possible errors, thereby preventing greater damage to the device [11], while also preventing a situation where the device cannot be controlled under regular maintenance.

## 2. Related Works

To improve the maintenance process through fault diagnosis, both the literature on optimization and the prevention of scheduled maintenance were addressed. Among all, predictive maintenance was the most promising and widely discussed topic in the different fields. With the application of novel innovative techniques, predictive maintenance has achieved better performance than conventional preventive approaches. There are several technologies used for predictive maintenance such as the model-based approach with the application of artificial intelligence (AI) and a data-driven approach, among others [12,13]. Some of these studies are mentioned in the following subsections. 

### 2.1. Predictive Maintenance with AI (Model-Based Approach)

With the development of AI and IoT, business in all industries can be reimagined. Collected data are not only used to analyze the past but also to predict the future. The most popular AI techniques for machine diagnosis are artificial neural networks and evolutionary algorithms. 

Marichal et al. [12] proposed a fault detection system based on AI techniques. In this paper, vibration characteristics of a marine oil separation system were analyzed. The obtained vibration signals were processed in the frequency domain and later used in a genetic neuro fuzzy system to predict the incipient faults.

Samhouri et al. [14] proposed an intelligent machine condition monitoring and diagnostic system using an adaptive neuro-fuzzy inference system (ANFIS) and a neural network system (NN) to predict fault types. The signal features obtained from the machines were used as inputs for both ANFIS and NN systems to predict the fault type.

Mushiri et al. [15] proposed an AI-based maintenance model for the petroleum storage industry. They used the Nowlan–Heap risk analysis matrix procedure to identify equipment which could potentially have complex problems; pumps tanks, valves, and diesel generators were identified as the critical equipment. A simplified pump diagnostic system was developed where the user has to provide inputs and then the expert system will provide a solution to the problem occurring in the pump. The AI-based intelligent system also provides maintenance for other equipment using sensors. 

Gou et al. [16] discussed the problem of the fault diagnosis of electric motors based on power signals and a genetic algorithm to optimize support vector machine (SVM) to obtain the optimized classification model. The implementation of fault diagnosis can recognize the operation status of the electric motor in time and, thus, a reaction is made accordingly to improve its reliability.

Syafrudin et al. [17] proposed a real-time monitoring system which provides an alert in advance for fault detection. This system is able to process and analyze large amounts of data effectively as it comprises IoT-based sensors, big data processing, and a hybrid prediction model. Here, the hybrid prediction model consists of noise-based outlier detection and random forest classification which can detect fault more accurately. It showed that the proposed model helped to improve decision making and prevent unexpected faults. 

Vianna et al. [18] proposed a method to identify degradation and future estimates subject to multiple wear conditions. Then, the results were integrated into a maintenance planning optimization algorithm based on extended Kalman filter and a multiple model technique for aeronautical redundant systems.

Jung et al. [19] proposed a novel analytical method to support the remaining usefulness lifetime estimation. It supports the predictive maintenance for optimizing the replacement scheduling over the equipment. The proposed analysis algorithm provides an accurate prediction which can avoid unnecessary expenses. 

### 2.2. Predictive Maintenance with a Data-Driven Approach

Data-driven approaches for fault detection and predictive maintenance are usually based on data analysis and statistical techniques like machine learning algorithms. Predictive models can be built based on enormous amounts of historical data via training. Through model testing and verification, such approaches are helpful in making reasonable predictions about the occurrence of any failures or the remaining lifecycle of a device in the future. 

Nabati et al. [20] proposed sources for how product use information (PUI) can be useful for wind energy. The PUI sources consist of radio frequency identification (RFID), sensor data, signals from condition monitoring, supervisory control and data acquisition (SCADA) systems, and historical data regarding maintenance which are used in maintenance activities. After data collection from these sources, decision making was conducted via data analysis. The outcomes of the data analysis contained suggestions which helped in the maintenance strategy of offshore wind power farms.

Patil et al. [21] predicted device failures by presenting the log information of a device using a data-driven approach based on machine learning. It reduced machines breakdown, improved customer satisfaction, and also reduced the cost for the original equipment manufacturers. This approach could predict failures in the components approximately 14 days earlier than the occurrence of actual failure.

Sipos et al. [22] proposed a data-driven approach based on a predictive model utilizing state-of-the-art machine learning techniques. This approach is based on multiple-instance learning for predicting equipment failures. Historical data regarding the equipment with log information were used to gather useful information which was then later used in predictive maintenance.

Rodseth et al. [23] presented a structured approach for data-driven predictive maintenance in terms of profit loss indicator. The outcomes of this study suggested that a data-driven maintenance strategy had a positive effect on profit loss indicator values which can be analyzed for long-term sustainability. 

Chen et al. [24] proposed a data-driven approach to identifying the conditions of an airplane with a self-sensing wing structure integrated with a sensor network. In this study, a large number of features were obtained on the basis of time, frequency, and information and a new filter feature selection algorithm was developed. Then, machine learning was applied which was helpful in real-time flight condition monitoring. 

Susto et al. [25] proposed a flexible maintenance scheduling decision-making system. In this study, machine learning and a regression algorithm was used to extract more features from the industry to calculate the remaining useful life. 

Borgi et al. [26] proposed a data-driven approach for predictive maintenance to detect the accuracy error of robot manipulators. For this, a correlation was established between the electrical signals measured from the robot and its accuracy values without any historical data. The results obtained from the predictive model were highly accurate and useful. 

Summarizing the above literature, it seems that many researchers use various techniques, including AI, smart data analysis, data-driven approach, etc., to achieve good results for predicting failures or for predictive maintenance. Condition-based data-driven approaches are becoming more widely used for early detection of anomalies. In this paper, we proposed a data-driven approach based on condition monitoring combined with data analytics. Usually, in data-driven predictive maintenance, one of the key components of the whole approach is the data itself; it should be vast and contain records of damage periods in addition to normal operation of the device. In this perspective, many of the data-driven approaches cannot be adopted in cases where no failure data are available in the historical data. Again, in some studies, only one feature was taken into consideration for predicting failures, such as the vibration analysis of the device. But predictive maintenance systems rely on a myriad of sensor data which contain information about other related factors for device condition monitoring. Thus, in this study, we proposed a data-driven condition monitoring approach which contains historical data collected from the experimental platform with vast, but without historical failure, data. We considered seven different kinds of variables containing environmental factors and also some features obtained from the device, including temperature, wind speed, sound, humidity, and vibration, etc. Depending on the machine, all of these factors also have an important role related to the impact on predictive maintenance. For example, an increase in temperature can lead to components melting or burning, and vibration analysis can offer insights into possible breakdowns with increased vibrations. Thus, depending on the system, maintenance can be scheduled before significant damage occurs. To do this, a correlation was initially established among these continuous variables in the experimental platform. It is noted that the load in previously mentioned variables can sometimes be designated as a situation to be monitored. In this paper, the load reflects the amount of air intake of the device and, simultaneously, a certain degree of dust accumulation. 

Most devices or industries require skilled persons to regularly monitor and evaluate the condition of equipment via sight or on-site collection of data such as temperature, sound, vibration, etc. Nowadays, however, it may challenging processes all data manually, where human resources are a problem. Thus, in this study, we proposed an intelligent predictive maintenance method where data can be collected and monitored through remote sensing. Although the whole system is not automatic, there is no need to be present on-site for data collection or monitoring. Then, according to the outcome of the predictive analytics, we can postpone maintenance if the device’s condition is healthy or we can preschedule maintenance if the need is urgent. Therefore, the purpose of this study was to quickly and effectively determine when the next maintenance session is required and to make model corrections and real-time predictions. Big data analysis requires long-term data accumulation and it is not easy to carry out repeated experimental verifications on any equipment. Therefore, to promote the idea of predictive maintenance proposed in this study, we used a dehumidifier as the base of the experimental platform and different kinds of sensors to obtain the working state of the device. Then, using SAS analytical tools, a one-to-one corresponding relationship was obtained among the monitored variables. The prediction results of this study were based on historical data and in addition to improve the prediction results the underlying factors also added to the analysis. The underlying factor was the most influential variable on the load. Thus, by manipulating the underlying factor, future changes in the predicted load can be observed and, also by changing the predicted load, the changes in the underlying factor can be observed. Therefore, it contributes more insight into maintenance prediction and we can predict the feasibility of maintenance. The built model achieved better target accuracy over time and, from the prediction results, the future load changes can be effectively controlled. 

## 3. Proposed System

### 3.1. System Architecture

The proposed predictive maintenance platform of this study was divided into two parts: sensing module and analysis system. The sensing module was mainly used to collect and upload the operational situations of the test platform for further monitoring and analysis. The analysis system was capable of quickly and effectively analyzing the accumulated data and making model corrections for failure predictions. Considering the real-time sensing data and the historic recorded data, it provides future maintenance guidelines for the test platform. 

#### 3.1.1. Sensing Module

The sensing module was composed of a Raspberry Pi (Adafruit, New York City, NY, USA) and PIC18F4525 microcontroller (Microchip technology Inc., Taipei, Taiwan). To achieve good results, the control kernel in the proposed system required Wi-Fi connectivity, the capability of integrating multiple sensors, online storage, and, more significantly, a powerful central processing unit (CPU) for the preprocessing of collected data for analysis. In order to achieve these requirements, we brainstormed the appropriate components, as there are different kinds of prototyping boards available on the market with different features and usages. It was found that in many studies, a Raspberry Pi and PIC microcontroller act as control kernels of systems and efficiently provide the features similar to the above discussed requirements [27,28,29,30]. Thus, in this work, the Raspberry Pi and PIC18F4525 were chosen as the control kernel. Raspberry Pi is a fully functioned single-board computer, a system-on-chip (SOC) device that runs on a Linux operating system and which can easily be switched by just swapping the memory card. It can do multiple tasks at the same time, much like a computer. Networking, data transmission, databases, and webservers can all be involved in Raspberry Pi-based applications. Also, it can be accessed remotely via Secure Shell. Raspberry Pi has no on-board analog-to-digital conversion (ADC) interface. To provide an ADC feature, the PIC18F4525 was selected. Due to the fact of its low cost, small-scale or micro-scale entrepreneurs can take advantage of the proposed Raspberry Pi and PIC18F4525 combined control kernel for real-world applications. 

**Figure 1:**
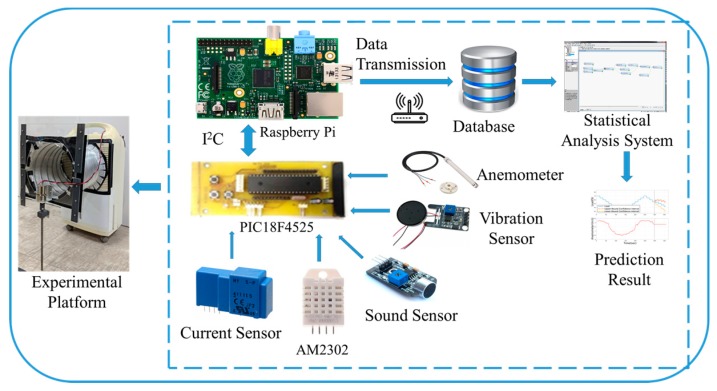
Overview of the proposed framework.

**Figure 2:**
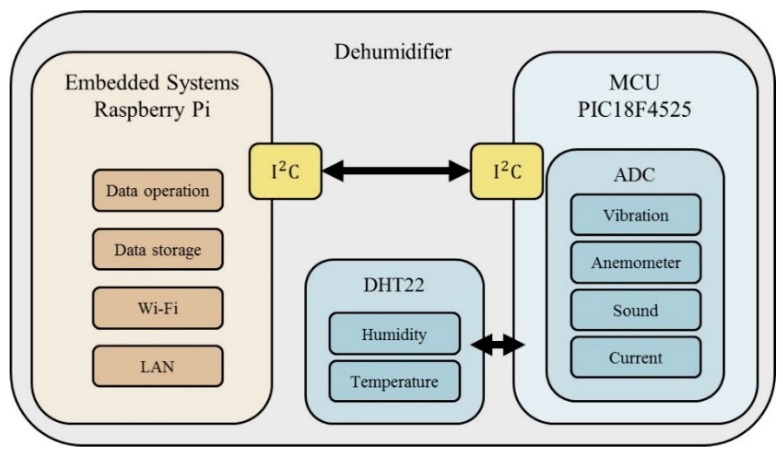
Sensing module architecture.

**Figure 3:**
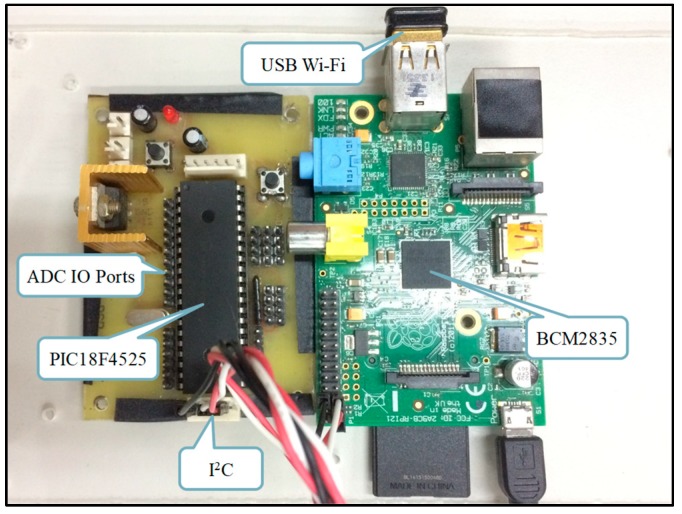
Image of the actual sensing module.

Different kinds of sensors are used according to the required sensing information. Then, the Raspberry Pi collects all the data from sensing nodes via wire or wireless networks, performs the data preprocessing, and uploads the data to the database for cloud analysis. The overview of the proposed framework is shown in Figure 1, the sensing module architecture is shown in Figure 2, and an actual image of the sensing module is shown in Figure 3.

##### A. Control Kernel

Raspberry Pi is basically a credit card-sized single-board computer developed in the United Kingdom by the Raspberry Pi Foundation. In this paper, we used Raspberry Pi v2 (Adafruit, New York City, NY, USA). One useful feature of the Raspberry Pi is the general-purpose input–output (GPIO) interface. The signal input or output is performed through the I/O pins. It has an ARM BCM2835 processor running with 700 MHz 512 MB RAM with an SD slot, an HDMI port, a composite video out, 3.5 mm audio jack, two USB ports, and an ethernet jack [31]. It allows a number of programming languages to be used by the users, such as Python, C++, C, and Java. The communication interfaces include I2C, UART, SPI, Wi-Fi, and LAN.

##### B. Sensors

In this work, the sensing environment selected in this paper was a dehumidifier and the dehumidification function was turned off in order to simulate a ventilation-like system. In Table 1, a list of typical industrial devices is provided to show which parameters should be monitored for effective predictive maintenance [8]. Here, five sensors and one sensing module were selected. As most of the sensors are analog types, they reduce the module volume and installation complexity. For analog-to-digital conversion, we used a low-cost PIC, which reduced the overall cost of the sensing module. Simultaneously, the wind speed, vibration, sound, current, ambient temperature, and humidity were monitored and the ADC was also conducted at the same time. Then, the requirements for collecting the equipment’s parameters were completed. The sensors used in this study are discussed below in details.

There are many types of anemometers available on the market with different features, such as cup- or rotational-type anemometers that measure both wind speed and direction. In addition, hot wire or thermal flow anemometers measure both the wind speed and pressure. In this work, the hot wire anemometer (FTS07, Eyc-Tech, New Taipei City, Taiwan) was used, where a wire was electrically heated in order to reach a certain degree above room temperature. When the air flows over the hot wire, there exists a linear relationship between the rate at which the wind is flowing and cooling of the wire; then, by measuring the heat loss of the wire, wind speed is calculated. As it needed to be installed in the experimental platform, a small-sized anemometer was selected.

In addition, a piezoelectric vibration sensor (605-00004, Parallax, Rocklin, CA, USA) was used to measure the vibration generated from the device. It has an LM358 chip as an amplifying circuit to adjust its sensitivity. The response sensitivity is high enough that its output can be adjusted by tapping via one’s hands. The working principle of the piezoelectric vibration sensor is to utilize the piezoelectric effect of the piezoelectric crystal; that is, the output charge signal of the piezoelectric vibration sensor is proportional to the external force. However, originally in the experimental platform it was found that the vibration generated by the operation of the device was very weak. Through the principle of vibration, it is generally possible to identify the presence or absence of sound but not possible to detect the intensity of sound. Thus, an additional sensor was required to be installed. 

Hence, a sound sensor (PKM13EPYH4002, muRata, Zhongxiao E. Road, Taipei, Taiwan) was added. In general, a sound sensor is used for sound-activated lamps or for cases of sound control or sound detection. However, in this device, when the intake air flow changes, significant sound signals which can be detected by this sound sensor are produced. 

A current transducer (HY 5-P, LEM USA Inc., West Bradley Road, Milwaukee, WI, USA) was also used to measure the current changes with respect to the variations of the intake air flow. In this work, this sensor was selected due to the fact of its compact design and for a wide current rating range.

Another sensor was used for the measurement of environmental temperature and humidity. In this work, the AM2302 sensor (Aosong Electronics Co., Ltd., Qingdao, China) was selected to measure both temperature and humidity to determine the impact of environmental factors on the device.

#### 3.1.2. Analysis System

**Figure 4:**
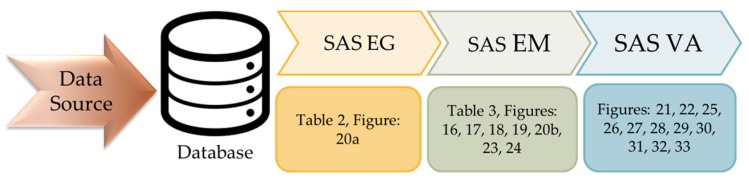
Analysis system architecture (SAS-Statistical Analysis System, EG–Enterprise Guide, EM–Enterprise Miner, VA–Visual Analytics).

For data analysis, the SAS (Statistical Analysis System) software suite developed by the SAS Institute was adopted. The analysis system used in this paper consisted of SAS EG (Enterprise Guide), SAS EM (Enterprise Miner), SAS VA (Visual Analytics), and a database. The architecture of the analysis system is shown in Figure 4. Different sets of data collected by the sensing module are stored in the database. After the data sorting and processing by the SAS EG, it is going to train and build models through SAS EM. Then, some user-friendly graphical reports with time series prediction are provided via SAS VA.

In this paper, SAS EG was mainly used for data conversion and correlation analysis. Users can easily import and open a sas7bdat. formatted dataset in SAS EG. Common CSV-formatted data can be transformed into sas7bdat. files via SAS EG along with data pre-processing. The data pre-processing contains data cleaning, transformation, and data editing such as the name, label, type, and filling missing data. In data cleaning, unproductive data which do not contain useful information are removed and, for missing data, the average value of the immediately available pre- and post-missing values is calculated to fill in the missing data. Afterwards, correlation analysis was carried out among the variables of the experimental platform. In this work, Pearson correlation analysis was used for analysis, where the strength of bivariate correlations can be observed. 

The SAS EM simplifies the cumbersome data mining process into five steps: data sampling, exploration, modification, modeling, and assessment to assist in the systematic and step-by-step process of data mining. It is used to unearth valuable information that has been deeply buried in the vast amounts of recorded data. In addition, SAS EM has an easy-to-use interactive interface to view the mining process and present the application steps in a flow chart, providing an intuitive and smooth data mining experience. In this paper, SAS EM was used for model comparison and model training. After the conversion of the multiple sets of experimental data through SAS EG, the roles of the selected variables were adjusted while importing. Among the multiple sets of experimental data, 70% of data were used for training the model and the remaining 30% of the data were used for model verification. Then, three different types of algorithms, including logistic regression, decision tree, and neural networks, were considered for performing model comparison. 

In this paper, SAS VA was used for underlying factor analysis to identify important variables and relationships among the data. First, it sets up and sums all the data attributes and finds the average in order to make subsequent prediction and analysis correct. The names, data types, model types, and formats can be set here.

### 3.2. System Implementation and Execution

#### 3.2.1. Experimental Platform Design

**Figure 5:**
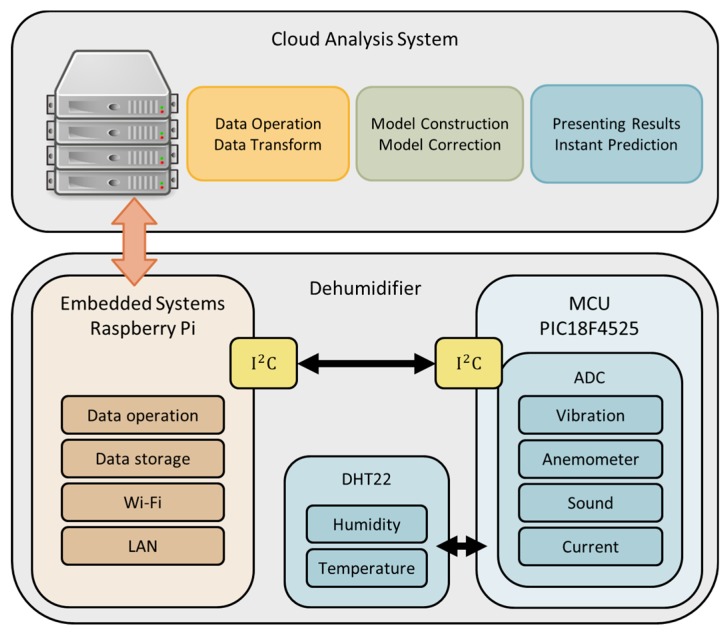
Experimental platform architecture.

**Figure 6:**
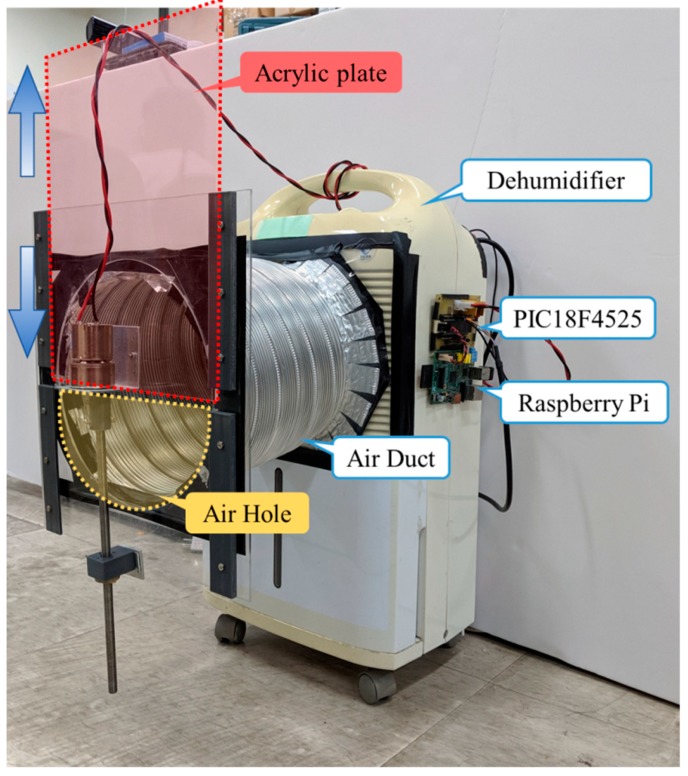
Real image of the experimental platform.

Practically, big data analysis requires long-term data accumulation and it is not easy to repeat the experimental verification. Thus, in this paper, to perform the repeated experiments and verifications, alternatively, a dehumidifier was adopted as the experimental platform with necessary modification. The architecture and the actual image of the experimental platform are shown in Figure 5 and Figure 6, respectively. In Figure 6, an acrylic plate moved up and down at a constant speed to, respectively, change the volume of air intake. When the acrylic plate was completely closed, it implied that the intake air flow was at a minimum level. Referring to Figure 6, the larger the air hole, the more intake air flow that is produced, which reflects the case where there is less dust accumulation. In summary, under this experimental setting, a larger amount of intake air flow simulates the case of less dust accumulation. Equivalently, a smaller amount of intake flow reflects the case having significant dust accumulation. In summary, the degree of dust accumulation can be emulated by controlling the size of the air hole. In this study, several sensors were installed in the dehumidifier such as wind speed, vibration, sound, current, temperature, and humidity to collect different kinds of data subjected to load variations. 

**Figure 7:**
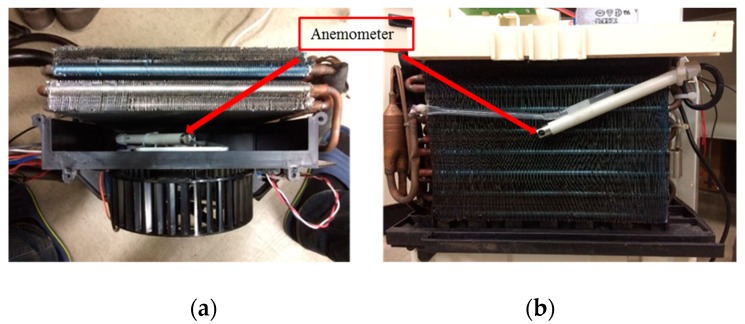
Anemometer installation on the equipment: (**a**) inside; (**b**) outside.

**Figure 8:**
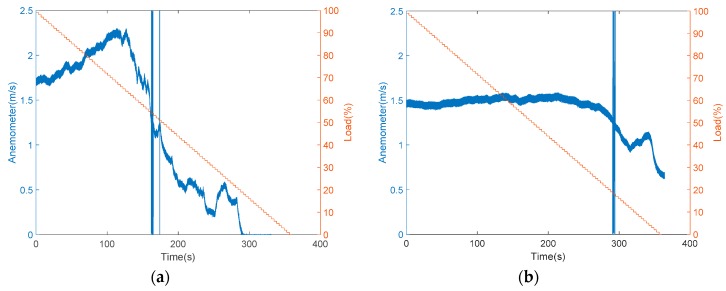
The measured wind speed versus load while the anemometer was installed: (**a**) inside; (**b**) outside.

**Figure 9:**
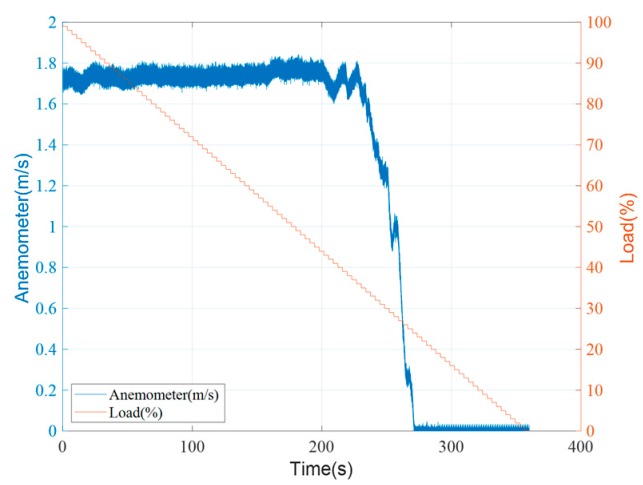
The measured wind speed with the air duct.

The anemometer was originally installed in two locations and tested separately to determine the difference between the inside and outside installations, as shown in Figure 7a,b, respectively. To install the anemometer inside, an experimental platform was needed to disassemble, shown in Figure 7a. In Figure 8 and the sequence figures, the variable load represents the volume of air intake that reflects the size of the air hole, which can be set by moving the acrylic plate up or down. During the experiments, the loads changed from 0% to 100%, meaning that the acrylic plate moved from the bottom to the top. After repeated tests, it was found that when the sensor was installed inside, there existed dramatic changes in the speed, between 70 and 120 s as shown in Figure 8a. This is because the sensor was too close to the drum blades, which led to instability in modelling and prediction. The straight lines shown in Figure 8a,b were due to the noise. Then, the anemometer sensor was installed outside, as shown in Figure 7b and the corresponding measurement data in Figure 8b. It was observed that the measured wind speed was relatively smooth. However, the wind speed did not converge to 0 m/s as expected when the acrylic plate was fully closed. According to existing studies, it was found that wind flow is hard to stabilize while air is entering through a narrow space in the equipment. In this paper, an air duct was installed between the equipment and the acrylic plate, and the results in wind–speed measurements are shown in Figure 9. It is obvious that the collected wind speed data were much smoother, and the response was quite fitting for the operational condition. 

**Figure 10:**
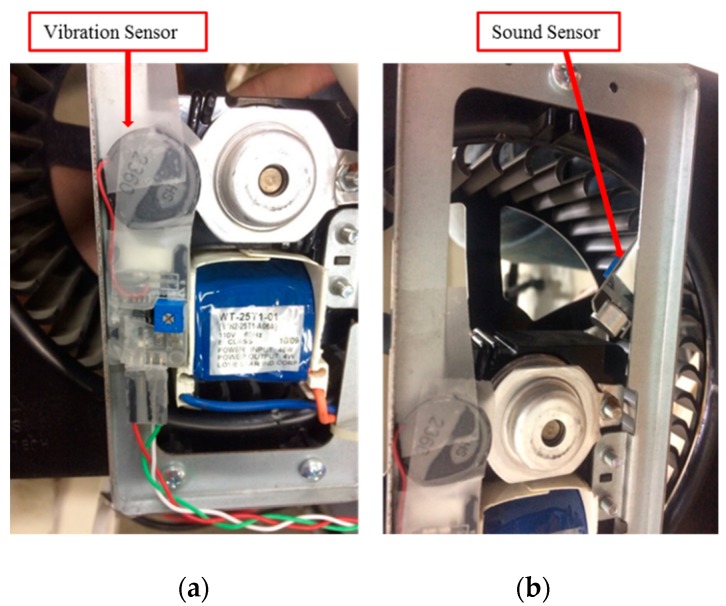
Sensor installation: (**a**) vibration sensor; (**b**) sound sensor.

**Figure 11:**
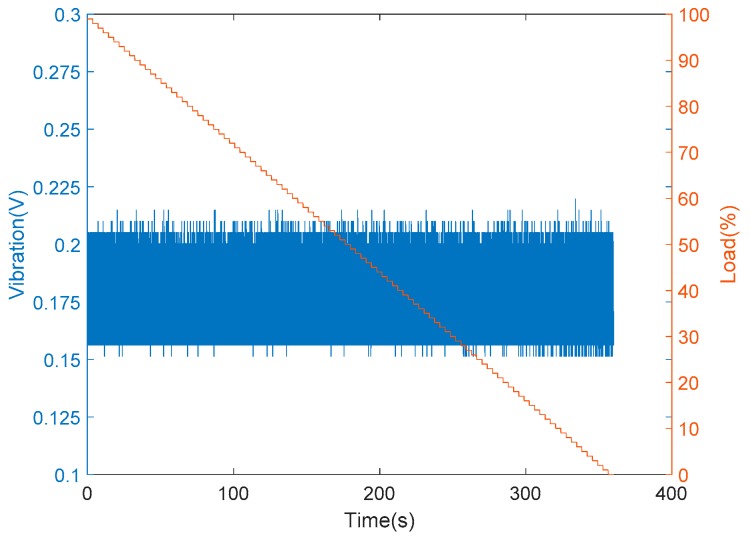
Experimental data from the vibration sensor.

**Figure 12:**
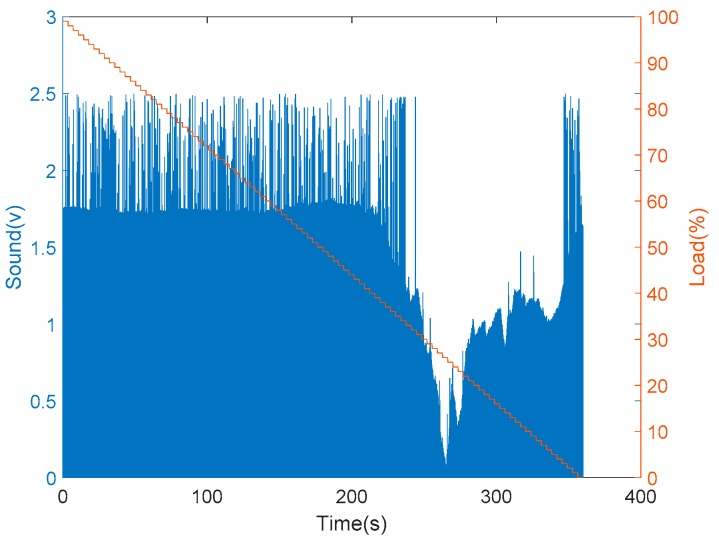
Experimental data from the sound sensor.

**Figure 13:**
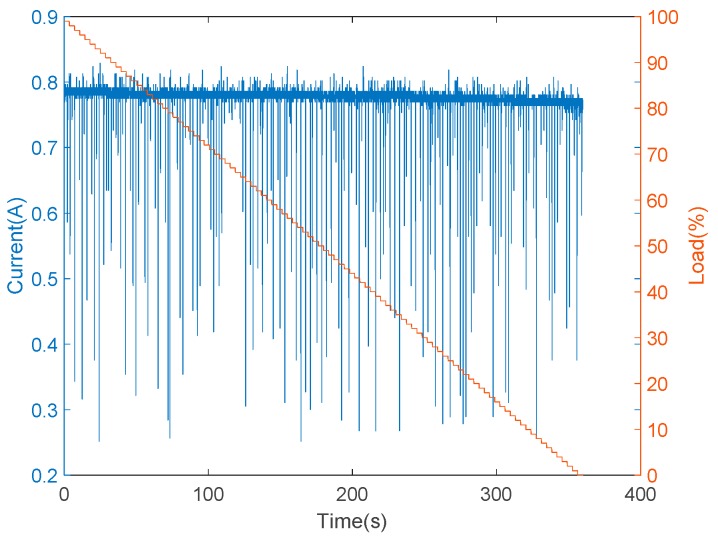
Experimental data from the current sensor.

**Figure 14:**
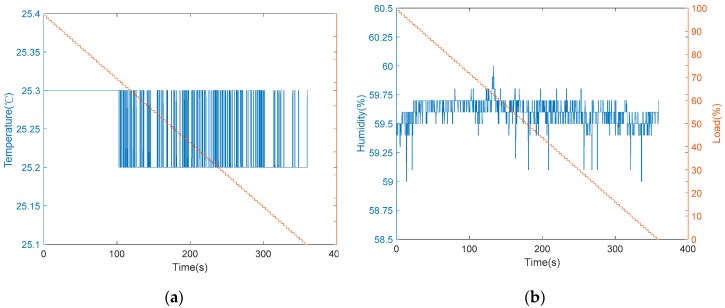
Sensing data versus load changes: (**a**) temperature sensor; (**b**) humidity sensor.

Furthermore, the installation of vibration and sound sensors are shown in Figure 10. The installation of vibration sensors was adjusted to generate the most sensitive responses, and the collected data with respect to load variations are shown in Figure 11. From Figure 11, it is found that there was no significant change occurred in the vibration sensor when the load varies. After many installation adjustments, the sensing responses still could not be improved upon and, therefore, a more sensitive sound sensor was installed as shown in Figure 10b. The data obtained from the sound sensor are plotted into a graph as shown in the Figure 12. From this signal graph it is clearly visible the difference between the sensed data of vibration sensor and sound sensor. After many experiments, it was found that the signal obtained was stable and changed linearly with the load. From the above test results, the sound sensor was selected instead of the vibration sensor and was provided to the analysis system for analysis and model construction. Similarly, the sensing data obtained from the current, temperature, and humidity sensors, installed in the device, are shown in Figure 13 and Figure 14, respectively. From the signal graphs, it was found that there was no significant change with respect to the load. This implies that these factors have less impact on the load as compared to other variables.

#### 3.2.2. Model Construction

**Figure 15:**
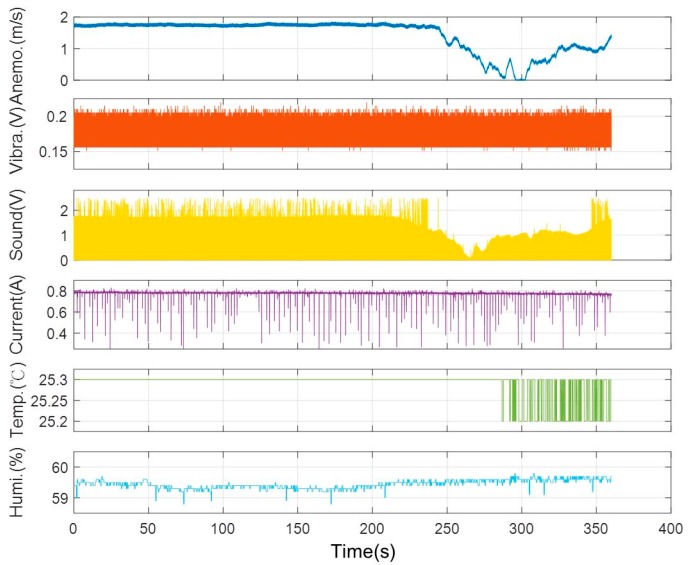
Data obtained from the experimental platform.

One example of the sensing data is shown in Figure 15. Data analytics was performed to analyze the correlations among the sensing variables, as shown in Table 2. The variable load was the size of the air hole as mentioned before. It means that when the acrylic plate was changed from completely closed to completely open, the load changed from the minimum to the maximum. In Table 2, it can be seen that the load corresponding to the anemometer had the highest Pearson coefficient value 0.75419, which means that the wind speed had the dominant impact factor and is considered as the underlying factor for the consequent validation and prediction tasks.

**Figure 16:**
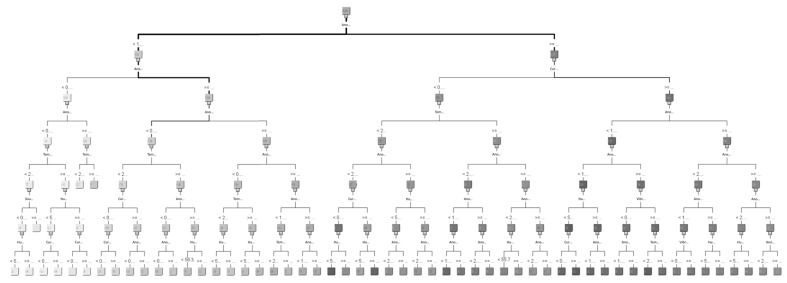
Decision tree model architecture.

**Figure 17:**
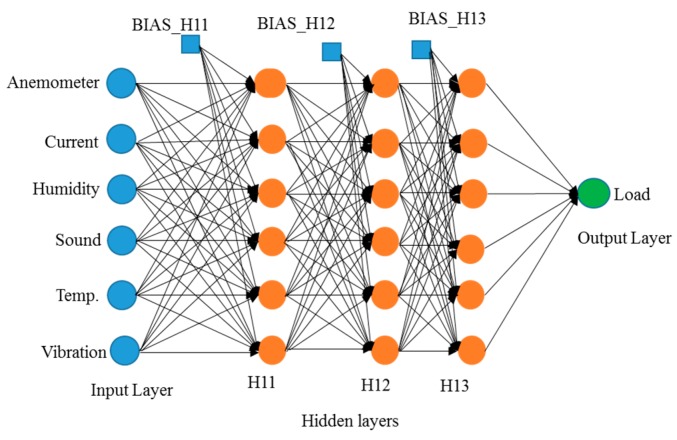
Neural network model architecture.

**Figure 18:**
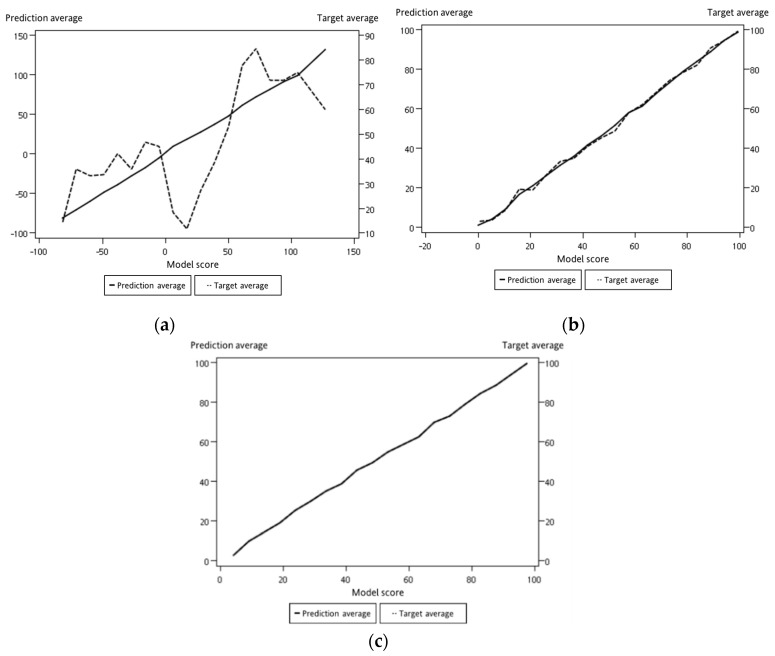
Model training and verification score map: (**a**) logistic regression; (**b**) neural network; (**c**) decision tree.

**Figure 19:**
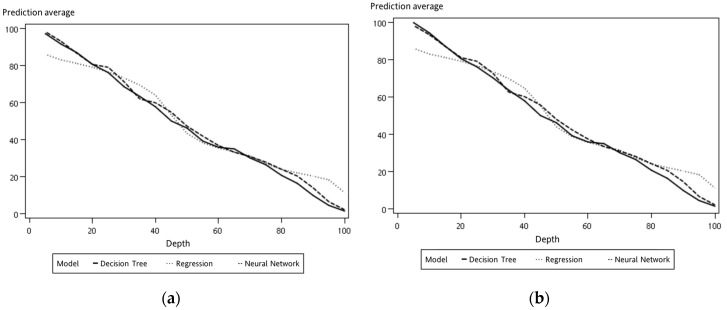
Predicted average of model comparisons: (**a**) training; (**b**) verification.

**Figure 20:**
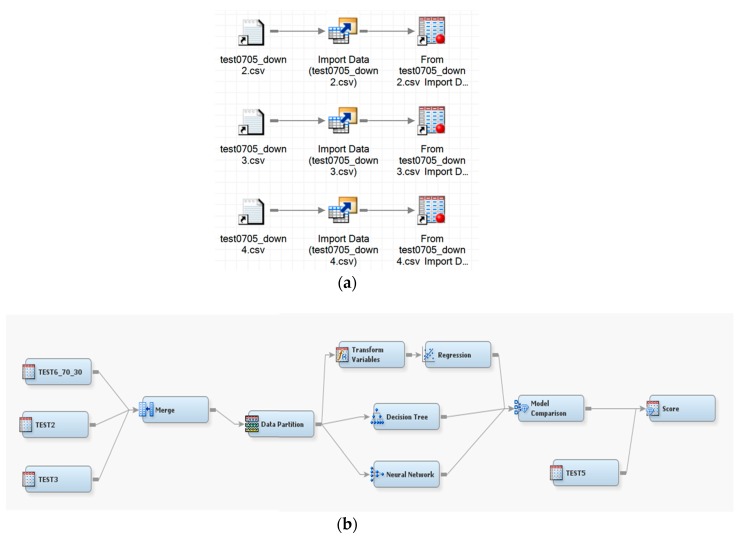
Process flow diagram: (**a**) data conversion by SAS EG; (**b**) SAS EM operation diagram.

For representing datasets, the process of model construction plays a vital role in data mining. Because it is important to determine which model is best fitted for the proposed work, in this study, three models for comparison were selected: logistic regression, decision tree, and neural network. Logistic regression is a statistical method used in data analysis where the dataset contains one or more independent variables. This method is appropriate when the dependent variable is dichotomous. It is used to find the best fitting model and to explain the relationship between the dependent variable and independent variables [31]. A decision tree is considered one of the best used supervised learning methods because it can construct models with high accuracy and stability. It is a flow chart that is similar to tree structure. It has a root, nodes, and leaves. The node, which has no incoming edges, is the root, the node with outgoing edges is called test node, and all other nodes are called leaves. In a decision tree model, each node represents a classification attribute, each branch represents a decision-making rule and each leaf represents the outcome of the target. The decision tree consists of a decision graph and possible outcomes that are used to create a plan to achieve the target value [32]. The architecture of the decision tree model is shown in Figure 16. The neural network is named after simulating a human neural model, using many connected artificial neurons to mimic the biological neural network. In general, there are three parts in a neural network: an input layer representing the input fields with units, one or more hidden layers, and an output layer representing the target. Here, each unit is connected with varying weights. So, the input values from the first layer are propagated to the output layers through the connected artificial neurons. This network learns through repeated trainings and each time it produces a prediction record and the network continues to improve its predictions until it will replicate the target criteria. Once trained, the network can be applied for unknown output prediction [33]. The architecture of the neural network model is shown in Figure 17. After the conversion of multiple sets of experimental measurements by SAS EG, collected data are imported to SAS EM. While importing the data, it adjusts the role of the variable and the selected target variable in a hierarchy. Multiple sets of data are combined and partitioned to choose 70% for model training and 30% for model verification. From the model comparison, a score table was obtained, as shown in Table 3. From the scoring observation of the analysis, the effective mean squared error of the three models were obtained. The above result indicates that the decision tree model had the lowest effective mean squared error, such that the decision tree was best suited to the device as a model, as shown in Figure 18 and Figure 19 and the work flow diagram of all these processes are shown in Figure 20. In Figure 18 and Figure 19, the target is designated as the pre-mentioned load variable. If the prediction average is more consistent with the target average, it means that the chosen model is more suited, corresponding to the testing data. In the following, the decision model will be adopted as the trained model for consequent prediction analysis.

#### 3.2.3. Data Prediction 

**Figure 21:**
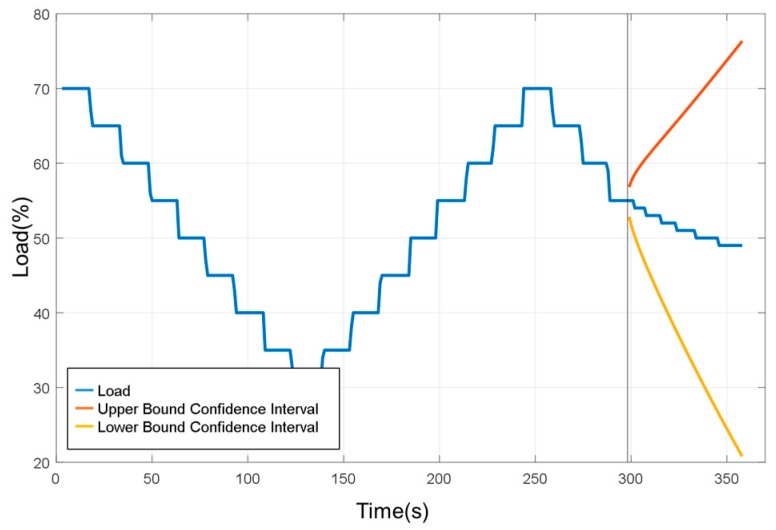
Prediction results (without underlying factor).

**Figure 22:**
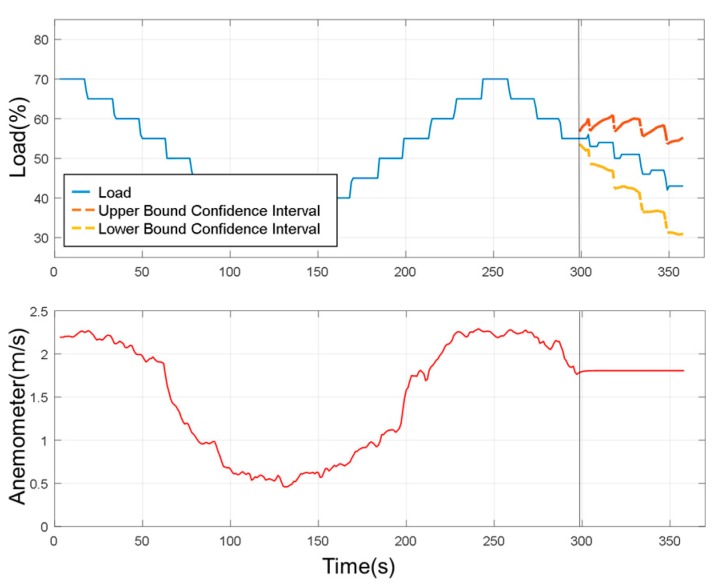
Predicting results (with underlying factor).

In this study, a one-by-multiple steps strategy was used for data prediction. It means one model is capable of predicting multiple outputs in one shot. When the experimental load goes back and forth from 70% to 30% over a period of time, SAS VA predicted the change in the confidence interval after 300 s for a period of 60 s without using the sensed experimental data as shown in Figure 21. When the underlying factor was added to the analysis, it was predicted under the wind speed factor. It can be seen that the range of the confidence interval was converging, as shown in Figure 22. It is reminded that the underlying factor addressed here was the wind speed, which was analyzed as the dominant factor corresponding to the degree of dust accumulation. It led to a smaller range in the confidence interval than the case without an underlying factor. Based on this observation, the underlying factor will be included for the load prediction.

## 4. Experimental Results and Analysis

**Figure 23:**
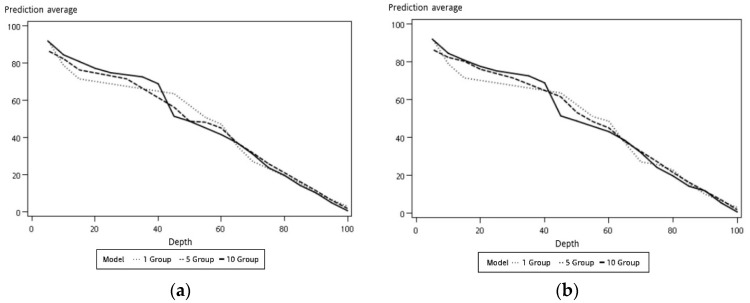
Model comparison versus different sets of data: (**a**) training; (**b**) verification.

**Figure 24:**
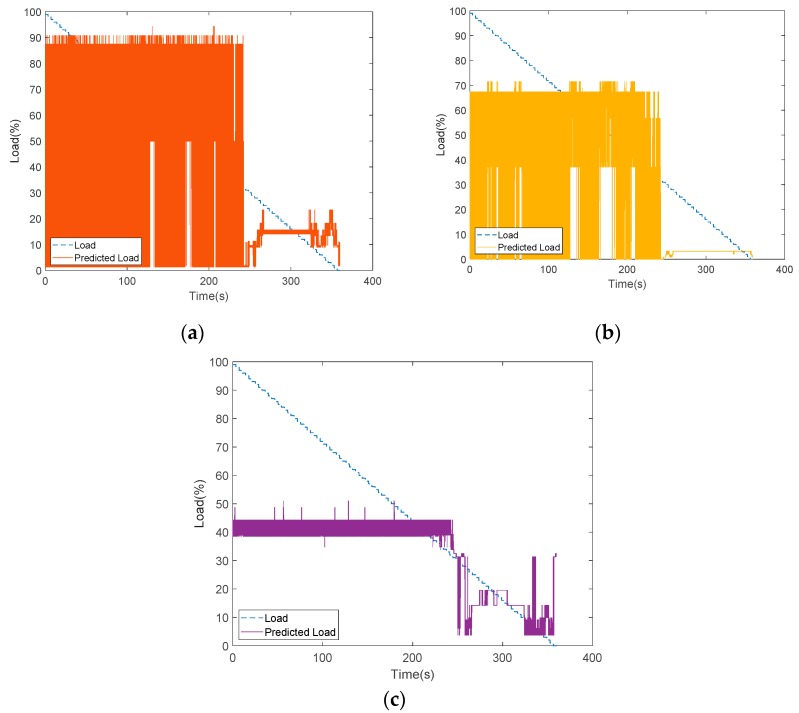
Prediction comparison with different sets of sensing data: (**a**) one set; (**b**) five sets; (**c**) ten sets.

As shown in Figure 15, the data collection time for each collected dataset from the experimental platform was 360 s. The experimental scenario as when the load decreased from 100% to 0%, i.e., the acrylic plate was changed from completely open to closed. In general, the accuracy of the model analysis can be improved using multiple sets of data. If a new set of data collected from the experimental platform is imported into the model, then the load estimation can be performed in real time. Here, we experimented by providing a new set of data as input to verify the model containing different sets of data, such as one set, five sets, and ten sets. With the decision tree model via SAS EM, it can be observed that the case with ten sets of data, the training and verification graphs were more consistent than the cases with fewer datasets, as shown in Figure 23 and Table 4. Similarly, the predictions of load variations corresponding to different sets of data are shown in Figure 24. It can be observed that with ten sets of sensing data, the load prediction was more consistent with the actual load when the load was less than 40%. In summary, the accuracy of load prediction can be improved with more sets of sensing data. Also, analyzing the results indicated that when the load was less than 40%, the prediction of the load had a certain degree of confidence.

In the Figure 19 and Figure 23, the *x*-axis parameter “depth” represents the percentile group in which the statistics were computed for each interval. The obtained score table from these outcomes shows that those values were calculated for every model for every partition of data for each interval, and the *y*-axis parameter “prediction average” represents the mean value calculated for each interval [34].

**Figure 25:**
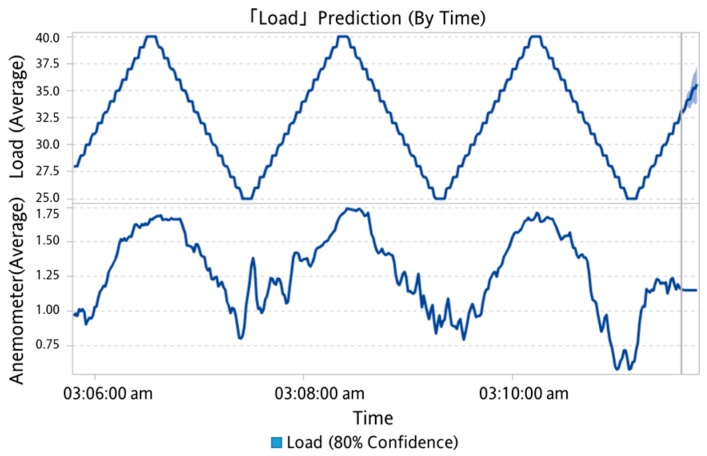
Prediction of future 10 s (with underlying factor).

**Figure 26:**
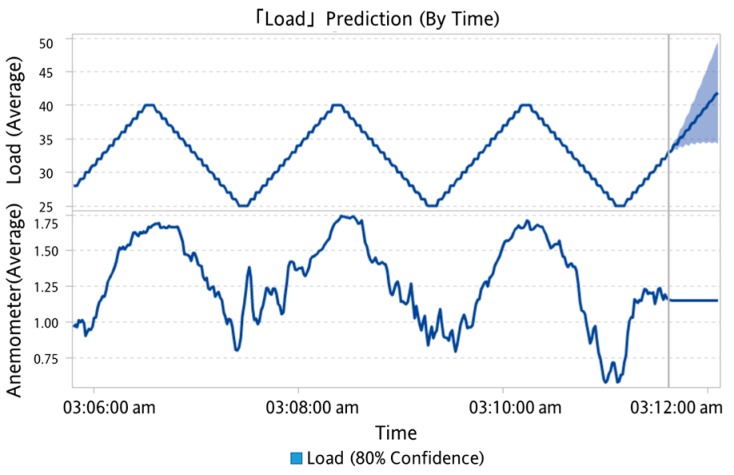
Prediction of future 30 s (with underlying factor).

To further verify the prediction results, the experiments were set to change the load coverage by 40% to 25% periodically to verify the prediction reliability. For better prediction, the analysis was done with the addition of the device’s underlying factor and the load condition was predicted for next 10 s and 30 s. Thus, the experiments predicted that the future load change interval for the next ten seconds without addition of device states would be much larger than the future load change interval with the device’s state added, as shown in Figure 25. This space indicates that when there is no underlying factor added under the fixed confidence interval, it is not easy to converge on future target trends. The prediction for the future 30 s is shown in Figure 26, where it can be observed that the area of the confidence interval was larger than the predicted 10 s in future, because the prediction time was longer. Although there was a fixed trend, it was still impossible to estimate for the long term.

**Figure 27:**
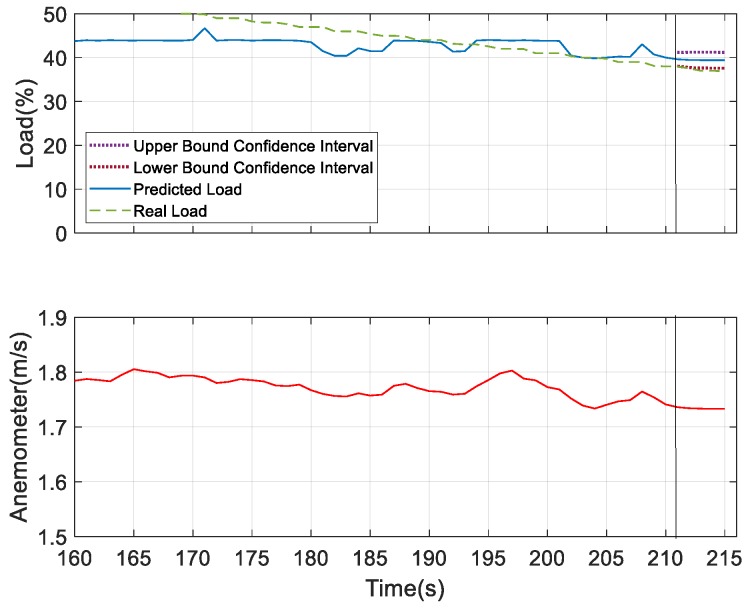
Load estimation and prediction for future 5 s.

As mentioned before, for better prediction, the analysis was performed with the addition of the underlying factor. The load condition was predicted for next 10 s and 30 s. As shown in Figure 25 and Figure 26, with all the same experimental settings, the confidence interval of the prediction of future 30 s was broader than the case for the future 10 s prediction. Then, the load prediction for the ten sets of the model, as shown in Figure 24c, was applied to SAS VA and also the anemometer data (underlying factor) was added, as it was the most influencing variable to the load. The results are shown in Figure 27. At 40% of the load, the prediction can be well observed and, at this point, the observed device degenerated. From the resulting graph, it was found that as the plate was gradually closed, the wind speed changed and the load value changed accordingly. Then the load estimation was predicted for the future 5 s.

**Figure 28:**
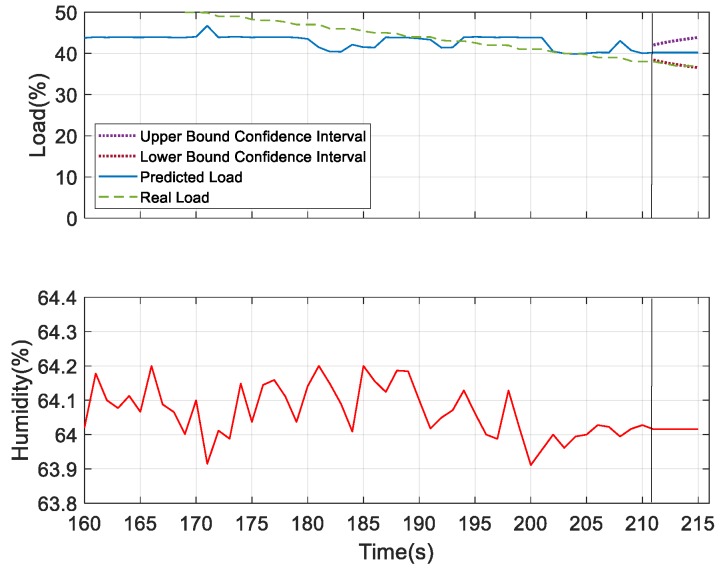
Prediction analysis considering humidity as the underlying factor.

**Figure 29:**
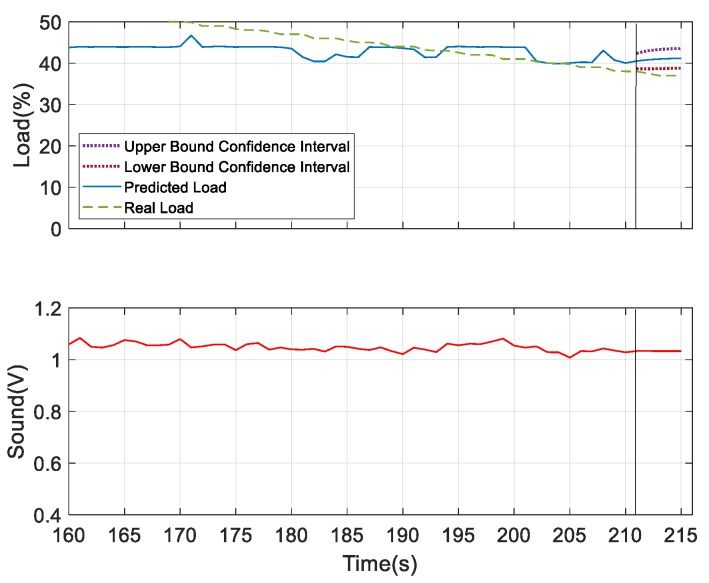
Prediction analysis considering sound as the underlying factor.

**Figure 30:**
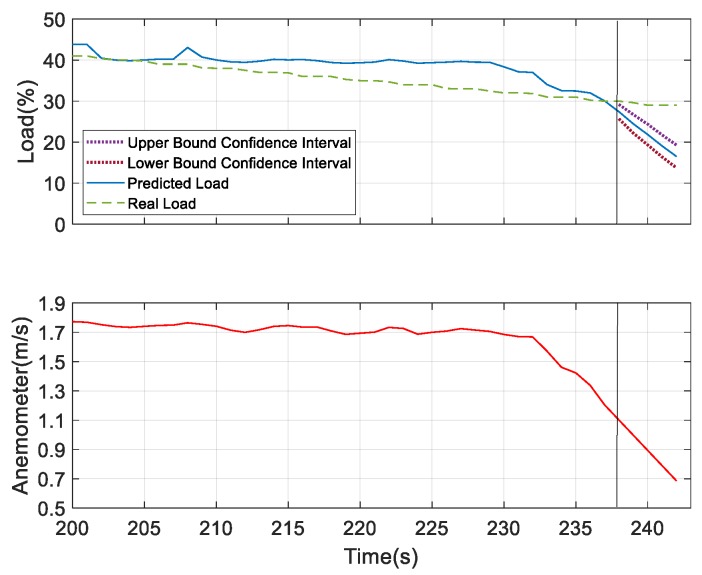
Prediction analysis at 30% of load considering wind speed as the underlying factor.

**Figure 31:**
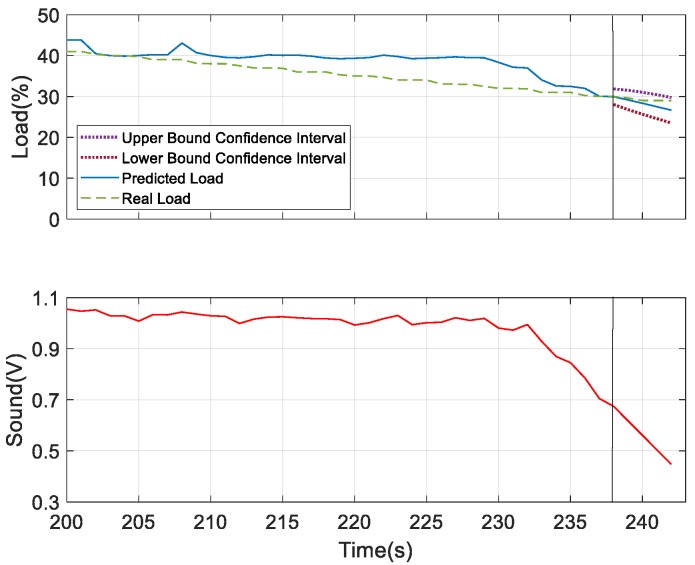
Prediction analysis at 30% of load considering sound as the underlying factor.

**Figure 32:**
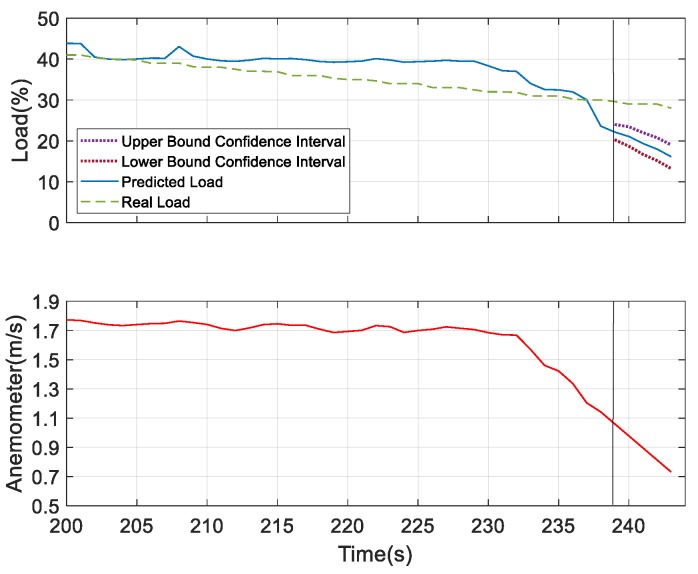
Prediction analysis at 25% of load considering wind speed as the underlying factor.

**Figure 33:**
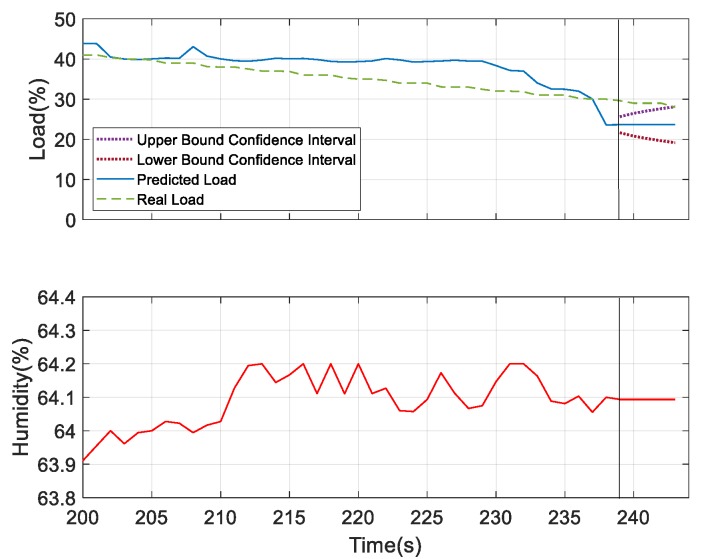
Prediction analysis at 25% of load considering humidity as the underlying factor.

For further verifying the prediction results, we considered humidity and sound as the underlying factors and compared the results with the most dominating underlying factor—wind speed. According to the obtained results for the prediction analysis, as shown in Figure 28 and Figure 29, it was observed that the confidence interval of the predicted load showed that wind speed was more convergent in nature compared to the other two. But the predicted load obtained was consistent with the real load in all the three figures, which indicates the better performing result. Thus, at 40% of the load, these three measures can be considered as the underlying factors. After this observation, we tried to verify the performance results based on the confidence interval and predicted load consistency with the real load assumed at 30% and 25% of the load. The more converged confidence interval with better consistency with the real load versus the predicted load was considered as better the performing result. At 30% of load for the prediction, we found wind speed and sound were the better underlying factors for prediction. But after comparing the obtained results of wind speed and sound as shown in Figure 30 and Figure 31, respectively, it can be seen that the sound variable had better performance than wind speed, as the predicted load was more consistent with the real load in the case where sound was considered as an underlying factor. So, at 30% of load, sound is considered as the underlying factor. Similarly, we verified the prediction result considering 25% of load. At this condition, wind speed and humidity were found to be better underlying factors for prediction. But after comparing both of the results obtained from wind speed and humidity, as shown in Figure 32 and Figure 33, respectively, the humidity variable had a better performance than wind speed, as the predicted load was more consistent with the real load. So, at 25% of the load, humidity is considered as the underlying factor. From these observations, it can be found that with different parameter settings, different outcomes can be obtained.

## 5. Discussion

First of all, each component was individually experimented with to analyze the results and performance. Then, all the components were integrated into the device for experimentation. Raspberry Pi was used to preprocess all the collected data and sent automatically to the database for future purposes. After, the data were assigned to SAS EG for data pre-processing, transformation, and correlation. During this process, the data were converted to the appropriate format for use in SAS EM to train the model. To improve the accuracy of the model, we tested different sets of data. Increased numbers of datasets based on the model provide better performance but consumes more time for computing the load as compare to a fewer number of datasets. After this, once a better performing model was selected, it was used for learning. Then, this model was used in SAS VA for prediction. So, it was not time consuming to compute the load during this step. Therefore, from the results obtained from the analysis system, we can predict the future condition of the device. Thus, the future breakdown of the device can be prevented by performing the required maintenance at an early stage. In this work, we used a dehumidifier as an experimental platform just to promote our method used for predictive maintenance within a short period of time. Thus, the developed technique was efficient as well as quite accurate; therefore, it can be applied to other real-world physical devices for prediction. Again, as SAS VA was used for prediction analysis, the underlying factor selection was not conducted manually; rather, the most influential variable was selected by the system automatically according to the settings. With these current settings, the system-selected underlying factors were wind speed, humidity, and sound but not the temperature, and if the parameter settings are changed, then the system may choose another variable as an underlying factor and conduct prediction analysis. Referring to Table 2, Pearson correlation analysis was conducted considering the whole dataset and found wind speed had the highest correlated value with the load. Thus, overall, wind speed was considered the best underlying factor. But during prediction at 40%, 30%, and 25% of load, it used a part of the dataset. Accordingly, the underlying factor also varied. Therefore, this feature may empower the research results. In future, we can further consider how to reduce the user’s operational burden and obtain faster future device status results. It is also possible to add a distributed operation from the front-end sensing module, which is directly used as a part of data conversion and processing. Thus, it can reduce the computing data of the backend server. In recent years, there has been much research discussing the management of equipment. In the future, we will be able to join network notification or mobile phone instant notification functions to the back end in order to achieve the goal of promoting predictive maintenance.

## 6. Conclusions

In our modern era, technology continues to change at the end-to-end of the business, optimizing its operations and not only considering preventive maintenance but also considering advantage of predictive maintenance tools to reduce down time, improve safety, and increase profits. With a data-driven approach along with machine learning, we have the ability to process massive amounts of sensor data faster than many other methods. This study combined the Raspberry Pi and the microcontroller PIC18F4525 as the core of the sensing module. The I2C communication protocol was used as the communication bridge between the two control cores, and the device’s status measured by the sensing module was accumulated. It was able to grasp the current usage of the equipment and provide an analysis system. After SAS analysis of these data, the correlation among multiple variables was obtained. The data that were more effective for the target and, thus, used for predictive maintenance was also found. The model proposed in this paper shows the feasibility of predictive maintenance. The constructed model can help to instantly check a device’s condition as well as future prediction with an improved target accuracy over time. Also, in terms of prediction, it is possible to observe future changes in the target from experimental results of the equipment.

## Figures and Tables

**Table 1:** Parameters monitored for predictive maintenance [8].

		Vibration	Humidity	Ambient Temperature	Environmental Pressure	Acoustic Signal	Thermography	Current
Device								
Pump		✓		✓	✓	✓	✓	✓
Valve			✓		✓	✓		
Motor/Fan		✓		✓		✓	✓	✓
Heat Exchanger		✓	✓	✓	✓			
Cable and Connector				✓			✓	

**Table 2:** Pearson correlation analysis.

Pearson Correlations, *N* = 161,999 Prob > |r| (Located H0): Rho = 0							
	Current	Anemometer	Sound	Vibration	Humidity	Temp	Load
Current	1.00000	0.01775 <0.0001	0.01872 <0.0001	0.00422 0.0896	−0.02195 <0.0001	0.01712 <0.0001	0.06286 <0.0001
Anemometer	0.01775 <0.0001	1.00000	0.61268 <0.0001	0.03468 <0.0001	−0.28185 <0.0001	0.30185 <0.0001	0.75419 <0.0001
Sound	0.01872 <0.0001	0.61268 <0.0001	1.00000	0.56845 <0.0001	−0.16808 <0.0001	0.18279 <0.0001	0.46089 <0.0001
Vibration	0.00422 0.0896	0.03468 <0.0001	0.56845 <0.0001	1.00000	−0.00462 0.0632	0.00269 0.2786	0.00912 0.0002
Humidity	−0.02195 <0.0001	−0.28185 <0.0001	−0.16808 <0.0001	−0.00462 0.0632	1.00000	−0.04557 <0.0001	-0.31014 <0.0001
Temp	0.01712 <0.0001	0.30185 <0.0001	0.18279 <0.0001	0.00269 0.2786	−0.04557 <0.0001	1.00000	0.54038 <0.0001
Load	0.06286 <0.0001	0.75419 <0.0001	0.46089 <0.0001	0.00912 0.0002	−0.31014 <0.0001	0.54038 <0.0001	1.00000

**Table 3:** Model comparisons.

Model	Training Mean Squared Error	Verification Mean Squared Error
Decision tree	7.228	7.473
Neural network	23.732	23.499
Logistic regression	213.544	218.439

**Table 4:** Model comparisons with different sets of data.

Number of Sets	Training Mean Squared Error	Verification Mean Squared Error
1	119.657	121.782
5	87.037	88.112
10	59.181	59.603
